# Intervenção Percutânea da Artéria Coronária Principal Esquerda. Por que os Dados do Mundo Real são tão Importantes?

**DOI:** 10.36660/abc.20210236

**Published:** 2021-06-08

**Authors:** Vinicius Daher Vaz

**Affiliations:** 1 Hospital do Coração Anis Rassi GoiâniaGO Brasil Hospital do Coração Anis Rassi , Goiânia , GO - Brasil

**Keywords:** Intervenção Coronária Percutânea, Doença da Artéria Coronária, Angiografia/diagnóstico por imagem, Prevalência, Revascularização Miocárdica, Acidente Vascular Cerebral, Mortalidade

A prevalência estimada da doença da artéria coronária principal esquerda encontrada durante a angiografia diagnóstica é de 6% em séries publicadas. O entusiasmo por uma terapia menos invasiva do que a cirurgia de revascularização do miocárdio (CABG) para pacientes com doença da artéria coronária principal esquerda desprotegida (ULMCA) remonta aos anos 90. ^1^ Embora a contribuição da CABG na sobrevida de pacientes com doença de ULMCA seja inegável, nos últimos anos, vários autores têm demonstrado a segurança e eficácia da intervenção coronária percutânea (ICP).

Apesar das controvérsias a respeito da publicação de 5 anos do estudo “XIENCE *versus* Coronary Artery Bypass Surgery for Effectiveness of Left Main Revascularization (EXCEL)” ^2^ , quando se trata de desfechos duros, como morte e acidente vascular cerebral, nos últimos anos vários ensaios randomizados e não-randomizados demonstraram não-inferioridade ou mesmo superioridade da ICP em comparação à CABG. ^2 - 6^ Recentemente, esses dados foram compilados em duas meta-análises nas quais o seguimento de longo prazo não mostrou diferença significativa na mortalidade e na taxa de AVC entre ICP e CABG. ^7 , 8^ Além disso, dois desses ensaios randomizados com seguimento prolongado de longo prazo, de até 10 anos, demonstraram bons resultados sustentados da ICP, com taxas de mortalidade semelhantes às da CABG, respectivamente, 14,5% x 13,8% e 27 % x 28%. ^4 , 5^

Nesta edição dos Arquivos Brasileiros de Cardiologia, Grion et al., ^9^ apresentam sua experiência com ICP para doença de ULMCA em uma série consecutiva de 107 pacientes. ^9^ Esses dados são muito importantes para toda a comunidade médica envolvida no tratamento da doença arterial coronariana, à luz da escassez de dados sobre CABG ou ICP para doença de ULMCA em nossa região. Do ponto de vista da medicina baseada em evidências, os ensaios clínicos randomizados (ECR) são o “padrão ouro” para avaliar a segurança e eficácia dos agentes terapêuticos, ainda mais no complexo cenário de comparação de dois métodos de tratamento invasivos, tão distintos como a CABG e a ICP. Entretanto, registros e experiências locais, como o de Grion et al. ^9^ são importantes para fornecer todo o espectro de pacientes tratados no ambiente do mundo real e a possibilidade de avaliar se os tratamentos e resultados dos ECRs são realmente aplicados diariamente. Os rígidos critérios de inclusão e exclusão necessários implicam que as populações do estudo muitas vezes não são representativas dos pacientes encontrados na prática clínica. Por exemplo, nos estudos EXCEL e NOBLE, mais de um terço dos pacientes elegíveis foram na verdade excluídos e quase metade deles devido a condições clínicas que levam o cirurgião cardíaco ou cardiologista intervencionista participante a acreditar que o equilíbrio clínico não estava presente.

O presente estudo incluiu uma população do mundo real, geralmente excluída de ensaios clínicos randomizados com complexidade aumentada. Diabetes estava presente em mais da metade dos pacientes (57%) e a média de idade foi de até 69 anos. A fração de ejeção média de 53% é menor do que a dos ECRs e um maior número de stents foram implantados por paciente (3,9). Finalmente, o escore SYNTAX médio foi 46 ± 23, substancialmente mais alto do que os dos estudos EXCEL, ^2^ NOBLE ^3^ e PRECOMBAT. ^4^ Apesar desses perfis de risco altamente clínicos e de lesão, eles alcançaram resultados muito bons em curto prazo com alto sucesso do procedimento (99%) e baixa taxa de mortalidade intra-hospitalar (1,86%). Taxas semelhantes foram observadas em nosso país por Constantini et al., ^10^ em 2011 (mortalidade intra-hospitalar de 1,4%), bem como nos principais registros internacionais do tipo “ *all-comers* ” ^11^ como DELTA 1, DELTA 2 ^12^ e MAIN-COMPARE, ^13 , 14^ onde o a mortalidade hospitalar foi de 2,0% e 1,1% e 0,8%, respectivamente. Apesar da relevância dos resultados intra-hospitalares, obviamente, os resultados do seguimento de longo prazo ainda são necessários para confirmar esses bons achados intra-hospitalares. Posto isto, devemos ter em mente que para alcançar bons resultados em longo prazo em qualquer tipo de intervenção para pacientes com doença arterial coronariana multiarterial estável ou ULMCA, é essencial ter uma mortalidade hospitalar abaixo de 2%.

Por outro lado, tem sido amplamente demonstrado que mesmo a ICP contemporânea, comparada à CABG, apresenta maior risco de revascularização repetida no seguimento em longo prazo. Nesse contexto, vale ressaltar a excelência do atual grupo, que utiliza o ultrassom intracoronário (USIC) para guiar a ICP em 100% dos pacientes com doença de ULMCA. Mesmo nos ECRs, a ICP guiada por USIC não excede 70% do uso. Além disso, há muita experiência e uma riqueza de evidências que apoiam o uso rotineiro de USIC na ICP da ULMCA. O uso de USIC durante a ICP da ULMCA é seguro e está associado a reduções substanciais em ECAM no seguimento de longo prazo, incluindo revascularização repetida e até morte. ^15^

Em conclusão, Grion et al. ^9^ demonstraram resultados hospitalares muito bons com a ICP da ULMCA complexa guiada por USIC em nosso ambiente. Conhecer e divulgar nossos resultados intra-hospitalares é o primeiro passo para podermos incorporar os resultados dos ECRs em nossa prática diária.
