# Supplementary material for: Intervenção Percutânea da Artéria Coronária Principal Esquerda. Por que os Dados do Mundo Real são tão Importantes?
Source: Arq Bras Cardiol. 2021 Jun 8;116(6):1109–10. [Article in Portuguese] doi: 10.36660/abc.20210236 (PMC8288549; doi:10.36660/abc.20210236)
Supplement: Translation

# Translation: Left Main Coronary Artery Percutaneous Intervention. Why are Real-World Data so Important?

**Authors:** Vinicius Daher Vaz

**Affiliations:** 1 Brasil Hospital do Coração Anis Rassi, Goiânia, GO - Brasil

**Keywords:** Intervention Coronary Percutaneous, Coronary Artery Disease, Angiography/diagnostic imaging, Precalenve, Myocardial Revascularization, Stroke, Mortality

The estimated prevalence of left main artery disease found during diagnostic angiography is 6% in published series. The enthusiasm for a less invasive therapy than coronary artery bypass grafting (CABG) for patients with unprotected left main coronary artery (ULMCA) disease dates back to the 90s.^1^ Although the contribution of CABG in the survival of patients with ULMCA disease is undeniable, in the last few years, several authors have demonstrated the safety and efficacy of percutaneous coronary intervention (PCI).

Despite the controversies regarding the 5-year publication of the Evaluation of XIENCE versus Coronary Artery Bypass Surgery for Effectiveness of Left Main Revascularization (EXCEL)^2^ trial, when it comes to hard outcomes, as death and stroke, in the last years several randomized and non-randomized trials have demonstrated non-inferiority or even superiority of PCI against CABG.^2 - 6^ Recently, these data were compiled in two meta-analysis where long term follow-up has shown no significant difference in mortality and stroke rate between PCI and CABG.^7 , 8^ In addition, two of these randomized trials with extended long-term follow-up, up to 10 years, have demonstrated sustained good results after PCI, with death rates similar to CABG, respectively, 14.5% x 13.8% and 27% x 28%.^4 , 5^

In this issue of *Arquivos Brasileiros de Cardiologia* , Grion et al.^9^ present their experience with PCI for ULMCA disease in a consecutive series of 107 patients.^9^ These data are very important for the entire medical community involved in the treatment of coronary artery disease, in light of the scarcity of data regarding CABG or PCI for ULMCA disease in our region. From the point of view of evidence-based medicine, randomized control trials (RCT) are the “gold standard” for evaluating the safety and efficacy of therapeutic agents, even more in the complex scenario of comparing two invasive treatment methods as distinct as CABG and PCI. However, registries and local experience, such as that of Grion et al.^9^ are important for providing the full spectrum of patients treated in the real world setting and the possibility of assessing whether the treatments and results of the RCTs are actually applied on a daily basis. The necessary strict inclusion and exclusion criteria implies that trial populations are often not representative of the patients encountered in clinical practice. For example, in the EXCEL and NOBLE trials, more than one-third of the eligible patients were actually excluded and almost half of them were so due to clinical conditions, which leads the participating cardiac surgeon or interventional cardiologist to believe that clinical equipoise were not present.

The present study included a real-world population, usually excluded from randomized clinical trials with increased complexity. Diabetes was present in more than half of the patients (57%) and the mean age was as high as 69 years old. The mean ejection fraction of 53% is lower than those from RCTs and a higher number of stents were implanted per patient (3.9). Finally, the mean SYNTAX score was 46 ± 23, substantially higher than those from the EXCEL,^2^ NOBLE^3^ and PRECOMBAT^4^ trials. Notwithstanding these hugely clinical and lesion risk profiles, they have achieved very good short-term results with high procedural success (99%) and low rate of in-hospital mortality (1.86%). Similar rates were observed in our country by Constantini et al.^10^ in 2011 (in-hospital mortality of 1.4%), as well as in the major all-comers^11^ international registries like DELTA 1, DELTA 2^12^ and MAIN-COMPARE,^13 , 14^ where the hospital mortality was respectively 2.0% and 1.1% and 0.8%. Despite the relevance of in-hospital results, obviously long-term follow-up outcomes are still needed to confirm these good in-hospital findings. Having said that, we must bear in mind that in order to accomplish good long-term results in any kind of intervention for patients with stable multivessel coronary artery disease or ULMCA, it is essential to have in-hospital mortality below 2%.

On the other hand, it has been widely demonstrated that even the contemporary PCI, compared to CABG, has a greater risk of repeated revascularization in the long-term follow-up. In this context, it is worth mentioning the excellence of the current group, using intracoronary ultrasound (IVUS) to guide PCI in 100% of the ULMCA disease patients. Even in the RCTs, IVUS-guided PCI does not exceed 70% of use. Moreover, there is plenty of experience and a wealth of evidence supporting routine use of IVUS in ULMCA PCI. IVUS during ULMCA PCI is safe and associates with substantial reductions in MACE in the long-term follow up, including repeated revascularization and even death.^15^

In conclusion, Grion et al.^9^ demonstrated very good in-hospital results of IVUS-guided complex ULMCA PCI in our environment. Knowing and publicizing our in-hospital results is
